# Investigation of CFRP-Countersunk Bolted Assembly Fatigue Damage under Three-Point Bending via Experimental and Numerical Analysis

**DOI:** 10.3390/polym15071648

**Published:** 2023-03-26

**Authors:** Zhengqi Qin, Ying He, Shengwu Wang, Cunying Meng

**Affiliations:** 1 School of Mechanical Engineering College, Dalian Jiaotong University, Dalian 116028, China; 2 School of Transportation and Electrical Engineering, Dalian Institute of Science and Technology, Dalian 116052, China; 3 College of Aerospace Engineering, Shenyang Aerospace University, Shenyang 110136, China

**Keywords:** fatigue damage, simulation, three-point bending, composite, countersunk bolt, preloading

## Abstract

In this research, the fatigue damage behavior under three-point bending of a composite joint incorporating a single countersunk fastener is investigated. Firstly, a self-developed fatigue test system was set up to test the fatigue characteristics of CFRP-countersunk bolted assembly under the displacement amplitude cycles of 10^3^ to 10^6^ to study the formation and expansion rule of damage and cracks. It found two typical damage processes, both of which involve some formal interface damage between fiber and matrix. Based on the experiment, a finite element fatigue damage analysis on this assembly was carried out according to the Hashin failure criterion. The simulation result shows an identical fatigue damage location and fatigue life with the experimental phenomenon. Moreover, it predicted the final fatigue life of the specimen under 10 hz cyclic loading with 1 mm displacement and 10 Nm bolt preloading. This research provides guidance for the engineering fatigue issues of single-bolted joint composite connection structures and provides a reference for the corresponding technical specifications formulation.

## 1. Introduction

Carbon-fiber-reinforced plastics (CFRP) have been wildly used in aerospace, automotive, wind power, civil construction, chemical, and other industrial fields; their application in aircraft has reached 50% of the total weight [1,2,3,4]. However, due to the limitations of materials and the manufacturing process, the traditional connection type cannot be used. In order to ensure the effective transmission of each component load, a reasonable connection method must be considered. As one of the most important connection types between composite materials, countersunk fasteners provide aerodynamic benefits compared to protruding-head fasteners [5]. However the countersunk hole can also cause the discontinuity of a structure, and it leads to non-uniform load distribution and stress concentration which makes the failure mode complicated under complex stress fields. Therefore, solving the fatigue damage problem of a countersunk hole has become a key issue to improve composite assembly components’ safety.

Previous studies have been carried out around the world in response to the above issues. In early years, D.S. Saunders et al. [6] summarized several types of fatigue damage around bolt holes, including hole ware and damage in the composite plates’ contact surfaces; Benchekchou [7] made a preliminary study on the static and fatigue performance of the composite laminates countersunk head bolt under bending load, analyzed the stress distribution around the bolted joint, originated a prediction method for crack initiation, and verified the theory with experiments. Fa Zhang et al. [8] investigated the quasi-static four-point bending behavior and failure characteristics of a large composite C-type beam with multi-bolt connections by numerical and experimental analysis and found that the bending load correlates with the ultimate compressive stress of the top composite skin result in beam failure. Zlatan Kapidžić et al. [9] proposed a bending moment calculation model for the CFRP countersunk bolt joint and proposed an s-n equation by the calculated bending stress amplitude, which has a good prediction fitting result with testing.

Smith and Pascoe [10] investigated the fatigue behavior of (0/90) carbon-fiber-reinforced plastic (CFRP) laminates with bolted joints under tensile loading. They found that the fatigue strength of the laminates was strongly affected by the initial clamping force and the bolt diameter, with larger bolt diameters and higher clamping forces resulting in increased fatigue strength. Herrington PD [11] investigated the fatigue behavior of composite bolted joints, including stress concentration and crack initiation and propagation. The results showed that the joint strength decreased with increasing stress concentration, and the fatigue life was sensitive to both the maximum stress level and the stress range. H.A [12] discusses fatigue evaluation of composite bolted and bonded joints. He shows that bonded joints have better fatigue performance than bolted joints, and the ultimate strength of composite bolted joints is related to the initial bolt torque. Ioannis K. Giannopoulos et al. [13] investigated the effect of bolt torque tightening on the strength and fatigue life of bolted joints in airframe FRP laminates. The study found that the torque value had a significant effect on the joint’s strength and fatigue life. Increasing torque values resulted in higher strength but shorter fatigue life, whereas decreasing torque values resulted in lower strength but longer fatigue life. It also proposed an optimum torque range that balanced strength and fatigue life. Christian Gerend et al. [14] proposed a new global progressive damage model for mechanical-fiber-reinforced polymers (FRP) under static and fatigue loading. It matches well with experimental measurements and observations in terms of static joint strength, cyclic stiffness degradation, and failure cycles.

These researches have partly announced the damage mechanization of CFRP-countersunk bolted joint structures under tension. However, there is a lack of research on the mechanical properties of the countersunk hole of CFRP under bending and the fatigue damage and failure process at the jointed position under bending cyclic loading. In particular, in certain engineering applications, such as the connection between drones and composite beams/ribs of composite wings, the structure load distribution is complex, and the force of the wing is based on the overall bending of the wing when the reciprocal distributed load acts. Therefore, it is of great engineering value to carry out research on the fatigue damage of CFRP-countersunk bolt assembly under cyclic load to guide aircraft design, manufacturing, and maintenance. This paper aims to explore the fatigue damage process of CFRP-countersunk joints under cyclic bending load using experimental and numerical methods. The experimental investigation was conducted using a self-developed 50t multi-axis fatigue electro-hydraulic servo test system, which is capable of performing a three-point bending fatigue test with a displacement loading of 10 Hz, and the numerical simulation was conducted using finite element analysis. The findings of this study will help to increase our understanding of the fatigue behavior of CFRP-countersunk bolted joints under cyclic bending loads and provide guidance for the design, manufacturing, and maintenance of aircraft structures.

## 2. Three-Point Bending Fatigue Test and Analysis of CFRP-Countersunk Bolt Assembly

### 2.1. Experimental Tests

#### 2.1.1. Fatigue Test System

**Figure 1:**
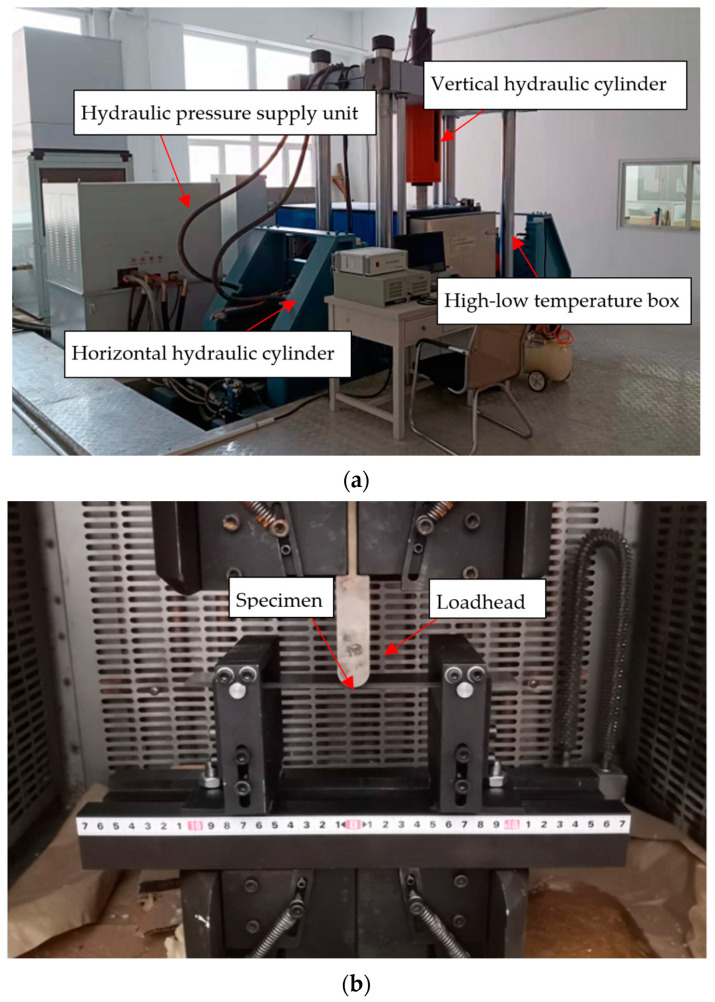

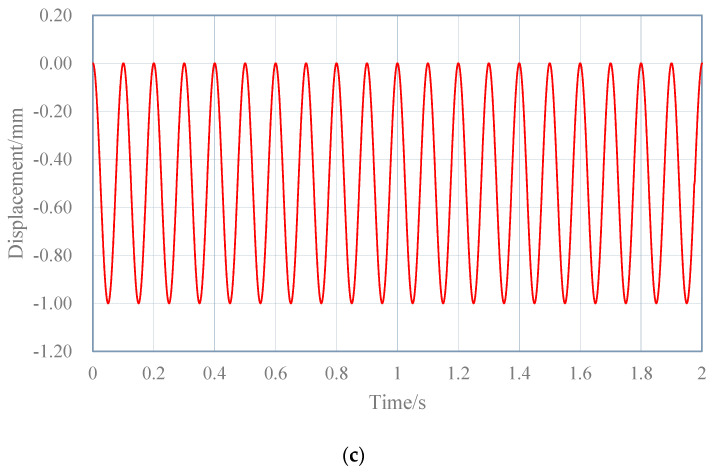
Test loading system and specimen installation. (**a**) Fatigue Test System. (**b**) Fixture and three-point bending specimen installation. (**c**) Displacement loading curve.

The study employed a self-developed 50t multi-axis fatigue electro-hydraulic servo test system, capable of conducting various tests such as uniaxial dynamic stretching, biaxial dynamic stretching, uniaxial tensile fatigue, and three-point bending fatigue tests, as presented in Figure 1a. The equipment is jointly developed by Shenyang Aerospace University and Shenyang Hongderun HVAC Equipment Co., Ltd., including a low-temperature chamber, a universal testing machine from Changchun Xintai Testing Machine Co., Ltd., and self-made three-point bending fixtures. The experimental fixture is completely self-developed and can better simulate the common ambient and low-temperature conditions of the specimen on the aircraft, ensuring the reliability of the test, illustrate as Figure 1b. A three-point bending fatigue test was conducted with a displacement loading of 10 Hz, where the minimum displacement was set to 0 mm, the maximum displacement was 1 mm, and the displacement amplitude was 1 mm, as illustrated in Figure 1b of the specimen fixture and Figure 1c of the loading curve. This test system enabled the researchers to accurately simulate the fatigue conditions that the material would experience in real-life applications, providing reliable data for the analysis of the material’s fatigue behavior.

#### 2.1.2. Test Specimens Preparation and Load Applying

**Figure 2:**
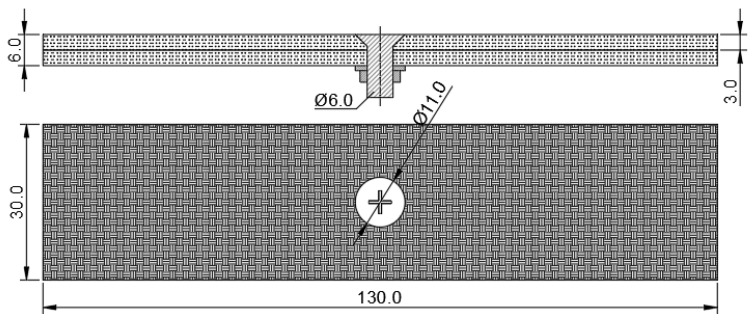
Countersunk bolt laminate specimen (Units: mm).

The plates of three-point bending fatigue specimens of CFRP-countersunk bolt assembly are made of T300BZ-3234 material, of 0/90° × 30 layers of plain weave composite carbon fiber prepreg, and are formed under high pressure in an autoclave at 0.5 MPa and 120 °C. The average thickness of the single-layer fiber woven fabric (including the matrix) is 0.1 mm, and the total thickness of the laminate sample is 3.0 mm. The size of the sample is 130 mm × 30 mm × 6 mm, and the countersunk head bolts are M6 bolts (according to the Chinese navigation mark “HB/1-129-2002 90° countersunk head bolts”) with a material of Ti_6_Al_4_V. A hole was drilled in the middle of the laminate, M6 countersunk head bolts were inserted, a steel washer with a thickness of 1 mm was placed between the nut and the laminate, and a preload torque of 10 Nm was applied to the bolt. The structure and size of the specimen are shown in Figure 2 (mm).

#### 2.1.3. Initial Processing Damage

Drilling the countersunk holes into the laminate with a bevel drilling tool. During the hole-forming process, because of low cohesive stress between layers of carbon fiber composites, the stress concentration may cause damage such as delamination, tearing, resin burns, etc. [15].

**Figure 3:**
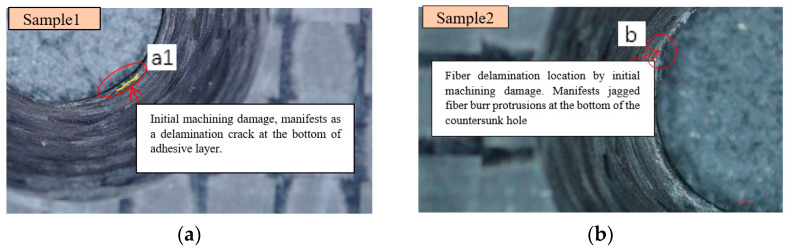
Micromachining damage before specimen loading.(**a**) Sample 1. (**b**) Sample 2.

Figure 3 shows two typical micro-damages in the oblique lower part of the countersunk hole due to the drilling opening. Although this picture is not clear enough due to the depth of field of the bevel, the microzone damage of sample 1 at region a1 can also be observed in Figure 3a. It manifests as a delamination crack at the bottom of the adhesive layer (hereinafter referred to as the initial delamination crack). The initial delamination crack is 1 mm in length, and the edge of the countersunk hole is smooth. Figure 3b shows the microdamage of Sample2 b location. It manifests as jagged fiber burr protrusions at the bottom of the countersunk hole. Both the two kinds of microdamage above will cause microzone stress concentration, which may cause the initial fatigue damage of the composite laminate.

#### 2.1.4. Specimen Fatigue Loading Damage

The specimens were loaded to 10^3^, 10^4^, 10^5^, and 10^6^ displacement cycles, respectively, and paused. The fatigue damage of the specimens was observed by an 80× optical microscope after unloading the bolts on the specimens.

**Figure 4:**
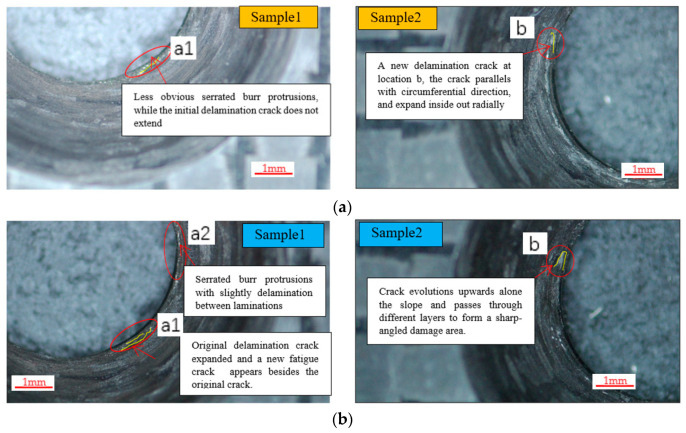

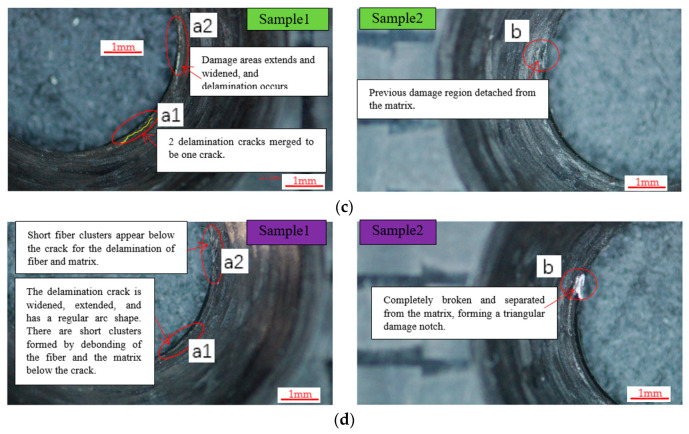
Fatigue damage. (**a**) Fatigue damage after 10^3^ cycles of loading. (**b**) Fatigue damage after 10^4^ cycles of loading. (**c**) Fatigue damage after 10^5^ cycles of loading. (**d**) Fatigue damage after 10^6^ cycles of loading.

In this study, the fatigue damage of Sample 1 and Sample 2 was observed after 10^3^ displacement cycles, and the results are presented in Figure 4a. Sample 1 shows wear on the countersunk hole edge, which is characterized by less obvious serrated burr protrusions. The initial delamination crack did not extend, and no changes such as crack widening were observed. On the other hand, Sample 2 generated a new delamination crack at location b, which parallels the circumferential direction and expands inside-out radially. The length of the crack was 1 mm.

The drilling micro damage showed that the fatigue crack formed and expanded at the location of the initial damage region of the CFRP-countersunk bolt hole. This could be due to the distribution of micro load concentration in this region. These findings suggest that the countersunk hole edge is a fatigue potential area, and further investigation is necessary to prevent or mitigate the damage in this area.

In Figure 4b, the fatigue damage of Sample 1 and Sample 2 is depicted after 10^4^ displacement cycles. For Sample 1, two types of damage were observed. Firstly, the original delamination crack at location a1 increased by 0.3 mm, and a new fatigue crack with a length of 0.5 mm appeared beside the original crack. The new crack is parallel in direction to the first original crack, as shown by Figure 4b region a1. Secondly, a new micro damage region a2 appeared on top of a1. The a2 region exhibits serrated burr protrusions with slight delamination between laminations and tearing through fibers on the hole edge.

For Sample 2, the delamination crack at b evolved upwards along the slope of the countersunk hole, passing through different layers and forming a sharp-angled damaged area. The two long sides of the damaged area were lifted, and a short side was connected with the material around the specimen hole, as shown in Figure 4b region b.

The observations indicate that the damage on Sample 1 has progressed significantly compared to Sample 2, with the appearance of a new fatigue crack and a micro damage region. On the other hand, Sample 2 exhibited delamination damage, which evolved upwards along the slope of the countersunk hole and formed a sharp-angled damaged area. The lift of the two long sides of the damaged area is likely due to the bending moment caused by the slope of the hole.

In Figure 4c, the fatigue damage of Sample 1 and Sample 2 after 10^5^ displacement cycles is illustrated. The observations indicate that the two delamination cracks in region a1 of Sample 1 have merged to form a single crack that evolves around the circumference. Moreover, the damaged area in region a2 has widened and extended, resulting in delamination. For Sample 2, region b is detached from the matrix, forming a discontinuous domain of material in the sharply angled damage region that appeared in the previous cycles.

The findings suggest that the fatigue damage in Sample 1 has progressed further, with the merging of the delamination cracks and the extension of the damaged area. The evolution of the crack around the circumference is also indicative of more severe damage. Similarly, in Sample 2, the formation of a discontinuous domain of material suggests a more severe level of fatigue damage.

In Figure 4d, the fatigue damage of Sample 1 and Sample 2 is depicted after 10^6^ displacement cycles. In Sample 1, the delamination crack in the a1 region widened and extended, forming a semi-arc regular shape along the circumference of the hole. Short fiber clusters appeared below the crack due to the delamination of fiber and matrix. In the a2 damage region, fibers in the layers and fibers between layers detached from the matrix, forming short fiber clusters. As a result, the bottom edge of the countersunk hole slope was damaged in a zigzag shape.

In Sample 2, the fibers in damage region b were completely broken and separated from the matrix, forming a triangular damage notch. The observed damage suggests that both samples experienced severe fatigue damage, with Sample 1 showing delamination and fiber/matrix detachment, whereas Sample 2 exhibited complete fiber breakage and matrix separation.

Previous investigation [16,17] shows that fatigue damage is usually accompanied by interface damage between fiber and matrix. In this study, the results are similar and indicate that the fatigue damage in specimens with initial machining damage around the countersunk hole under cyclic loading is characterized by the generation and expansion of interlaminar cracks, flake damage, and detachment caused by fiber breakage and matrix failure. Two typical types of damage were observed as follows, in order of preference:

(1) Serrated edges, fiber protrusions, delamination cracks, matrix failure, crack evolution, appears short fiber clusters, and final ply failure;
(2) Serrated edges, fiber protrusions, delamination cracks, cracks propagation through different layers, creaks evolution, forming flake damage, material detached, and final failure.

These observations indicate that fatigue damage is a complex phenomenon that involves multiple mechanisms, including interlaminar crack formation, matrix failure, and fiber breakage. The appearance of short fiber clusters and final ply failure suggests that the fatigue behavior of laminated composite materials is strongly influenced by the presence of machining damage, which can act as stress concentrators and increase the likelihood of crack initiation and propagation. Therefore, it is essential to carefully control the machining process to minimize the presence of initial damage and ensure the structural integrity and reliability of composite components.

### 2.2. Finite Element Model

FEM is an important method to study the mechanical properties of composite materials. The finite element analysis of composite materials can generally be processed by considering three calculation methods: the micro-scale approach, meso-scale approach, and macro-scale approach. Researchers such as P.A. Sharos [18] set up the FE model by a meso-scale approach to investigate the composite bolted joints at various loading rates. They did not set up the countersunk hole model, but captured a series of nodes by MPC. It simulates the bolted joint by BEAM element. This method was to be shown capable of producing high-fidelity simulations of joint behavior up to and including catastrophic failure, with CPU times being orders of magnitude less than that required for full 3D simulations. However, this method is not suitable for our study since it could not distribute the stress in and around the countersunk hole, and these locations are the main damage locations known from the experimental test above. Two studies [19,20] both built up a new element to the FE analysis of a single-lap, single-bolt composite bolted joint, and their methods both demonstrate an exact outcome. However, they did not consider the bolt and composite laminar contact, and this contact may be important for the countersunk bolted joints.

In this article, the main purpose is to investigate the fatigue characteristics of the composite countersunk bolted joints. Moreover, based on the experimental test above we find that a 3D model with a solid bolt is essential for our research.

#### 2.2.1. Geometry Model

**Figure 5:**
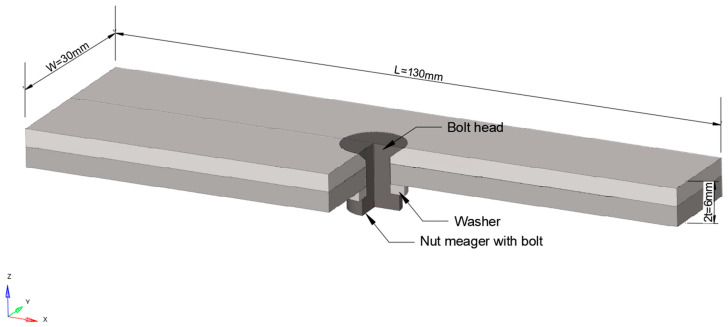
Schematic diagram of the geometry.

A 3D model was created for the same specimen as shown in Section 2.1.1, using a discrete rigid body of a fulcrum and loading indenter. The gasket had an inner diameter of 5.3 mm, an outer diameter of 10.06 mm, and a thickness of 1.5 mm. The fulcrum and loading indenter were modeled as discrete rigid bodies without consideration of their mechanical properties. The model is depicted in Figure 5. This approach was taken to simulate the mechanical behavior of the specimen under load, which allowed for the analysis of stress and strain distribution in the vicinity of the countersunk hole. The use of a discrete rigid body for the fulcrum and loading indenter simplified the model and reduced computational complexity.

#### 2.2.2. Material Properties

Table 1 and Table 2 provide a detailed list of the material properties of the countersunk head bolt, gasket, and composite laminas. These properties are essential in predicting the mechanical behavior of the components during cyclic loading and subsequent fatigue damage. In order to comprehensively describe the various damage situations that may occur in the fatigue damage process, the Hashin damage criterion was selected for the progressive failure analysis of composite materials [21]. This criterion is widely used in the aerospace and automotive industries for predicting the failure modes of composite structures subjected to fatigue loading. Additionally, Table 3 provides the friction coefficients of specific contact pairs [22], which are crucial in accurately modeling the contact behavior between different components during the fatigue test.

#### 2.2.3. Load and Boundary Conditions

Contact properties between composite laminates, bolts, and gaskets have a potent effect on simulation results, so this research performs a finite element analysis based on the contact conditions. Each contact pair uses a surface-to-surface contact, and considering the significant relative sliding between contact pairs during the fatigue loading process, the finite sliding contact type is reasonable.

Set 2 analyzes the FE model. Firstly, set preloading and secondly apply a certain displacement on the loading indenter.

Identical bolt preload torque is related to preload force according to the literature [23]:

(1) $$T=KF^{\prime }d$$

where *T* represents the preload torque. *d* denotes the thread diameter. *F*′ is the preload force. *K* is the tightening torque coefficient.

For M6 bolt, *d* = 6.02 mm. Moreover, in this paper, use *K* = 0.2. When *T* = 10 Nm, then the applied preload force can be derived according to Equation (1)

(2) $$F^{\prime }=\frac{T}{Kd}=8.3\;kN$$

Since the mode has symmetry, the structure is modeled by taking half of the total model and setting a symmetric boundary on the symmetric plane.

#### 2.2.4. Meshing

**Figure 6:**
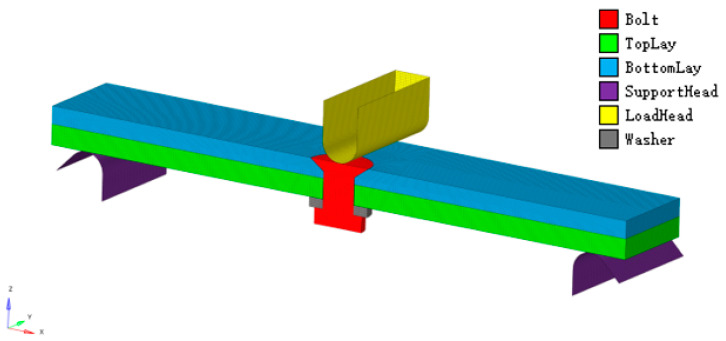
Simulate analysis FEA model mesh distribution.

The composite laminate elements’ type is SC8R with 0° along the x-axis and 90° along the y-axis and the global mesh size is 0.3 mm × 0.3 mm × 0.1 mm, where 0.1 mm is the element thickness alone z-direction. The countersunk bolt and gasket are meshed by the C3D8R with the size of 0.33 mm × 0.33 mm × 0.2 mm. The fulcrum and loading indenter is modeled as a discrete rigid body. There are a total of 562,264 elements and 602,256 nodes in this model. Figure 6 is the model grid division.

#### 2.2.5. Hashin Damage Criterion

In order to describe the various damage situations in the fatigue damage process comprehensively, select Hashin damage criterion for the progressive failure analysis of composite materials. Hashin 3D damage criterion predicts four failure types: fiber tensile failure, fiber compressive failure, matrix tensile failure, and matrix compressive failure. The fiber tensile failure is considered a shear effect; the simplification is formally shown as:

Fiber tensile failure (*σ*_11_ ≥ 0):

(3) $$F_{ft}=\frac{\sigma_{11}^{2}}{X_{T}^{2}}+\alpha \left(\frac{\sigma_{12}^{2}+\sigma_{13}^{2}}{S_{12}^{2}}\right)=1$$

Fiber compressive failure (*σ*_11_ < 0):

(4) $$F_{fc}={\left(\frac{\sigma_{11}}{X_{c}}\right)}^{2}=1$$

Matrix tensile failure (*σ*_22_ + *σ*_33_ > 0):

(5) $$F_{mt}=\frac{{\left(\sigma_{22}+\sigma_{33}\right)}^{2}}{Y_{T}^{2}}+\frac{\sigma_{12}^{2}+\sigma_{13}^{z}}{S_{12}^{2}}+\frac{\sigma_{23}^{2}-\sigma_{22}\sigma_{33}}{S_{23}^{2}}=1$$

Matrix compressive failure (*σ*_22_ + *σ*_33_ < 0):

(6) $$F_{mc}=\left[{\left(\frac{Y_{c}}{2S_{23}}\right)}^{2}-1\right]\frac{G_{2}+\sigma_{33}}{Y_{c}}+{\left(\frac{\sigma_{22}+\sigma_{33}}{2S_{23}}\right)}^{2}+\frac{\sigma_{23}^{2}-\sigma_{22}\sigma_{33}}{S_{23}^{2}}+\frac{\sigma_{12}^{2}+\sigma_{13}^{2}}{S_{12}^{2}}=1$$

where *X_T_* denotes longitudinal tensile strength, *X_C_* is longitudinal compression strength, *Y_t_* is lateral tensile strength, *Y_c_* represents lateral compression strength, and *S*_12_ and *S*_23_ are shear strength in the plane.

The equation upon is used to describe the damage interaction of composite material. For instance, *F_ft_* equals 1 represents the beginning of fiber damage initially along the tensile direction, and *F_ft_* less than 1 indicates that damage did not occur. This is the same as other damage types.

### 2.3. Result under Static Load

**Figure 7:**
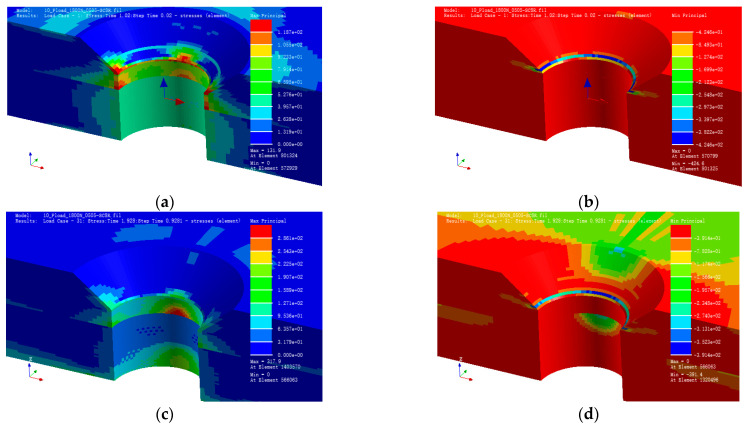
Max./Min. principal stress. (**a**) Max. principal stress. (**b**) Min. principal stress. (**c**) Max. principal stress. (**d**) Min. principal stress.

Figure 7a and b depict the preloading conditions. The maximum principal stress is located on the bottom edge of the countersunk hole with a value of 237.4 MPa, whereas the minimum principal stress is located at the maximum principal stress location with a value of −223.8 MPa.

Figure 7c and d illustrate the maximum and minimum principal stress under a three-point bending load with a displacement of 1 mm for the loading indenter. The maximum principal stress is 332.8 MPa, whereas the minimum principal stress is −265.7 MPa.

FEA verification confirms that the stress distribution nephogram above conforms to the law of composite fiber laminate stress distribution with a hole under preloading and bending loads. The stress distribution around the countersunk hole corresponds with the finding of Liu [24] and Egan [25]. They both investigated the stress distribution by numerical methods around the composite lamina hole and found that the distribution is correlated with the layering angle of the fibers around the hole. These results demonstrate the stress distribution characteristics of the specimen under different loading conditions, providing valuable information for the analysis of fatigue behavior and failure modes of composite structures with countersunk holes.

### 2.4. Fatigue Analysis

**Figure 8:**
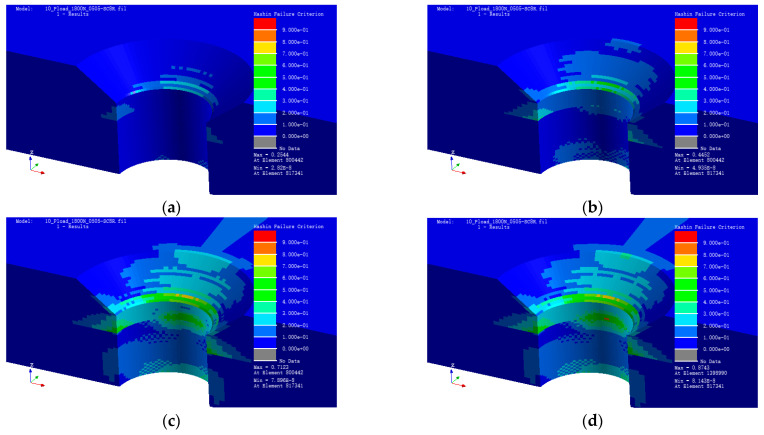
Laminate damage. (**a**) Laminate damage after 10^3^ cycles. Hashin damage value 0.254. (**b**) Laminate damage after 10^4^ cycles. Hashin damage value 0.445. (**c**) Laminate damage after 10^5^ cycles. Hashin damage value 0.712. (**d**) Laminate damage after 10^6^ cycles. Hashin damage value 0.874.

Using the FEA result above, simulate the assembly with a displacement fatigue load based on the Hashin damage criterion. The load is a sine wave with a frequency of 10 Hz, the loading time is 10^3^ s, 10^4^ s, 10^5^ s, and 10^6^ s respectively. The Hashin damage value is shown in Figure 8a–d.

**Figure 9:**
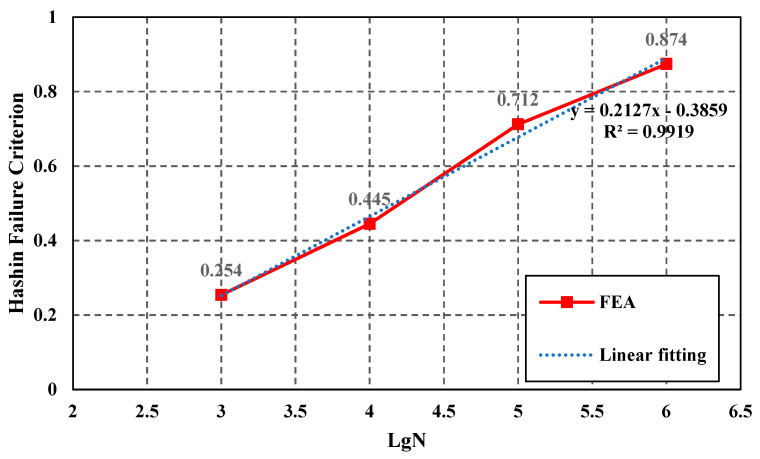
lgN-HFC correlation relationship.

According to the results presented in Figure 8a–d, the damage value on the bottom of the countersunk hole slope gradually increases with the increase of loading cycles. The location of the damage in the FEA results correlates with the location of the experimental sample. Based on the final fatigue results, a curve of cycle number lgN and Hashin damage value was plotted as shown in Figure 9.

The simulation results show that HFC has a linear relationship with lgN, which is the logarithmic load cycle number N (the dark solid line). The two parameters fit a linear curve (the light dash line) as shown in Equation (7); here, if HFC equals 1, the N ≈ 3.0 × 10^6^, which means that if the load cycles are 3.0 × 10^6^, damage occurs. Based on the simulated result, the final damage is located upon the countersunk hole bottom slope edge.

HFC = 0.2127 × lgN − 0.3859(7)

Regrettably, due to the limitation of machining, it is not currently possible to manufacture the hole in the laminate without initial machining damage. Moreover, in this FEA model, there was a failure to incorporate micro-damage and initial defects into the model. Imperfection is one of the focused solutions in further research.

## 3. Conclusions and Outlook

The main conclusions obtained in this study are summarized as follows:

(1) Based on the test results, the fatigue potential areas of CFRP-countersunk head bolt assembly are located upon the countersunk hole bottom slope edge. After 10^6^ cycling loading, obvious fatigue damage was observed on the specimen, and the primary formal damage is plying delamination caused by a matrix fracture, followed by fiber delamination on the countersunk hole slope, and fiber burr-like protrusions along the slope edge.
(2) The results of the finite element simulation based on the Hashin failure criterion show that the potential damage zone is located upon the countersunk hole bottom slope edge. At this time, the fatigue life is estimated to be 3 × 10^6^ load cycles. The simulation results are consistent with the experimental results in order of magnitude.
(3) A relationship between Hashin Failure Criterion and lgN was built up in this paper, where N is the number of cyclic loads. This equation shows a linear relationship between the two factors, but neglects the other factor as preloading, the hole diameter, and so on. Considering these factors is also one of the next research directions.
(4) This experiment is susceptible to the accuracy of machining and may also affect the results of the experiment to some extent. However, in this article, we do not consider the effect of clearance values, nor the influence of different preload states on material damage and life. Therefore, this aspect will be added in the next study.

## Figures and Tables

**Table 1:** Material properties of countersunk head bolts (Unit: N-s-MPa-mJ).

Parameter Name	Yang’s Modulus (GPa)	Poisson’s Ratio	Yield Stress (MPa)	Linear Expansion Coefficient
Countersunk bolt	110,000	0.29	950	1.2000 × 10^−5^
gasket	210,000	0.3	235	

**Table 2:** Material properties of T300BZ-3234 (Unit: N-s-MPa-mJ).

Parameter Name	Numerical Value	Parameter Name	Numerical Value
Yang’s modulus-11 (MPa)	69,000	Longitudinal tensile strength (MPa)	756
Yang’s modulus-22 (MPa)	69,000	Longitudinal compression strength (MPa)	557
Yang’s modulus-33 (MPa)	8300	Lateral tensile strength (MPa)	756
Poisson’s ratio-12 planeμ1	0.064	Lateral compression strength (MPa)	557
Poisson’s ratio-13 planeμ1	0.064	Longitudinal shear strength (MPa)	118
Poisson’s ratio-23 planeμ1	0.32	Lateral compression strength (MPa)	118
Shear modulus-12 plane (MPa)	4200	Longitudinal tensile fracture energy (mJ)	45
Shear modulus-13 plane (MPa)	4200	Longitudinal compression fracture energy (mJ)	0.6
Shear modulus-23 plane (MPa)	4200	Lateral tensile fracture energy (mJ)	45
		Lateral compression fracture energy (mJ)	0.6

**Table 3:** Friction coefficient of each contact pair.

Cantact Pair	Friction Coefficient
Composite-composite	0.3
Bolt-composite	0.1
Bolt-gasket	0.15
Gasket-composite	0.1

## Data Availability

The data that support the findings of this study are available on request from the corresponding author.

## References

[1] Ekh J., Schön J., Melin L.G. (2005). Secondary bending in multi fastener, composite-to-aluminum single shear lap joints. Compos. Part B.

[2] Hagnell M., Åkermo M. (2015). A composite cost model for the aeronautical industry: Methodology and case study. Compos. Part B Eng..

[3] Van Der Sypt P., Chérif M., Bois C. (2017). Analysis of the fatigue behaviour of laminated composite holes subjected to pin-bearing loads. Int. J. Fatigue.

[4] Galos J. (2020). Thin-ply composite laminates: A review. Compos. Struct..

[5] Chen X. (2016). Strength of Composite Sandwich Plate Fastened with Countersunk Bolts. Master’s Thesis.

[6] Saunders D.S., Galea S.C., Deirmendjian G.K. (1993). The development of fatigue damage around fastener holes in thick graphite/epoxy composite laminates. Composites.

[7] Benchekchou B., White R.G. (1995). Stresses around fasteners in composite structures in flexure and effects on fatigue damage initiation part 2: Countersunk bolts. Compos. Struct..

[8] Zhang F., Zhang W., Hu Z., Jin L., Jia X., Wu L., Wan Y. (2019). Experimental and numerical analysis of the mechanical behaviors of large scale composite C-Beams fastened with multi-bolt joints under four-point bending load. Compos. Part B Eng..

[9] Kapidžić Z., Granados D.L.Á., Arias J.A.M., Aguilera M.J.Q., Rodríguez J.P.C., Callejas J.C.G. (2022). Bolt fatigue in CFRP joints. Int. J. Fatigue.

[10] Whitworth H.A. (1999). Fatigue evaluation of composite bolted and bonded joints. J. Adv. Mater..

[11] Giannopoulos I.K., Doroni-Dawes D., Kourousis K.I., Yasaee M. (2017). Effects of bolt torque tightening on the strength and fatigue life of airframe FRP laminate bolted joints. Compos. Part B Eng..

[12] Smith P.A., Pascoe K.J. (1987). Fatigue of bolted joints in (0/90) CFRP laminates. Compos. Sci. Technol..

[13] Herrington P., Sabbaghian M. (1993). Fatigue Failure of Composite Bolted Joints. J. Compos. Mater..

[14] Gerendt C., Dean A., Mahrholz T., Englisch N., Krause S., Rolfes R. (2020). On the progressive fatigue failure of mechanical composite joints: Numerical simulation and experimental validation. Compos. Struct..

[15] Fan B., Chen Y., Chen B., Liang Y., Sun Y. (2019). Finite element analysis on heat distribution ratio during grinding CFRP. Diammond Abras. Eng..

[16] Tserpes K., Papanikos P., Labeas G., Pantelakis S. (2004). Fatigue damage accumulation and residual strength assessment of CFRP laminates. Compos. Struct..

[17] Zhao R., Chen Y., Li J., Liu S., Wang Y., Aung H.H., Kong X., Du B. (2023). Interface damage driven electrical degradation dynamics of glass fiber-reinforced epoxy composites. Compos. Sci. Technol..

[18] Sharos P.A., McCarthy C.T. (2020). Novel Finite Element for Near Real-time Design Decisions in Multi-fastener Composite Bolted Joints under Various Loading Rates. Compos. Struct..

[19] Belardi V.G., Fanelli P., Vivio F. (2019). FE analysis of single-bolt composite bolted joint by means of a simplified modeling technique. Procedia Struct. Integr..

[20] Liu F., Yao W., Zhao L., Wu H., Zhang X., Zhang J. (2020). An improved 2D finite element model for bolt load distribution analysis of composite multi-bolt single-lap joints. Compos. Struct..

[21] Hashin Z. (1980). Failure criteria for unidirectional fiber composites. J. Appl. Mech..

[22] Montagne B., Lachaud F., Paroissien E., Martini D., Congourdeau F. (2020). Failure analysis of single lap composite laminate bolted joints: Comparison of experimental and numerical test. Compos. Struct..

[23] Chen D. (2008). Handbook of Mechanical Design.

[24] Liu L. (2014). The Strength Research and Failure Analysis for Composite Joint Structure.

[25] Egan B., McCarthy C., McCarthy M., Gray P., Frizzell R. (2012). Modelling a single-bolt countersunk composite joint using implicit and explicit finite element analysis. Comput. Mater. Sci..

